# Optimum second call imputation in PPS sampling

**DOI:** 10.1371/journal.pone.0261834

**Published:** 2022-01-21

**Authors:** Fariha Sohil, Muhammad Umair Sohail, Javid Shabbir

**Affiliations:** 1 Department of Education, The Women University, Multan, Pakistan; 2 Department of Statistics, University of Narowal, Narowal, Pakistan; 3 Department of Statistics, Quaid-I-Azam University, Islamabad, Pakistan; Univerza v Mariboru, SLOVENIA

## Abstract

The current study deals with imputation of item non-response in probability proportional to size (PPS) sampling. A new imputation procedure is proposed by using the known co-variance between the study variable and the auxiliary variable in the case of quantitative sensitive study variable by considering the non-response in a randomization mechanism on the second call. An empirical study is conducted at the optimum values of *k*_*og*_ and *n*_*og*_ for the relative comparisons of ratio, difference, and proposed estimators, respectively, with the Hansen-Hurwitz estimator.

## 1 Introduction

Survey sampling is a technique which is utilizes in almost every field of life to estimate the finite population parameters with limited response. There are many sample selection procedures, which provide reliable data by selecting the representative sample. In equal probability sampling schemes, the probability of selection is equal for all the units in target population. If units varying in size, equal probability sampling may not give the appropriate importance to large or small units in the population. The appropriate importance to the population units is assigned by allocating the unequal probabilities of selection to the different units in the population. Thus, when units are different in size and variable under study is correlated with their auxiliary information e.g. size, then the selection probabilities may be assigned in proportion to their sizes. For example,

1. Colleges with large number of educational departments are likely to have more students and more faculty members. For the funds allocation, it may well be desirable to adopt a scheme of selection in which colleges are selected with probabilities proportional to their students or departments.
2. In an industrial survey, the number of workers may be selected as size of industrial area.
3. In biological studies, the number of patients may be selected according to the size of the hospital.

For all of these cases, the selection of sampling units is proportional to the size of auxiliary information associated with the particular unit, is called sampling with probability proportional to size (*PPS*). It is well known that the proper use of auxiliary information at estimation stage or at design stage or at both stages is helpful to magnify the performance of resultant estimators. Ratio, product, and regression estimators are good examples in this context.

In many real life situations, where non-response/refusals may affect the reliability and accuracy of data sets. These refusals are mostly occurred due to many reasons such as time of survey (during summer or winter vacations, office hours etc.), survey contents (embarrassing nature of questions, double barrel question etc.), respondent burden (irrelevant questions, length of questionnaire etc.), or data collection methods (telephone or mail surveys, personal interviews etc).

Initially, [1] provides an idea of sub-sampling the non-respondents of first call by dividing the population into two strata; respondents and non-respondents at first call. The detailed discussion on the proposed estimator is given in Subsections 1.1 and 1.2 for the case of simple random and PPS sampling scheme, respectively.

### 1.1 Sample selection in simple random sampling

Let Ω = {Ω_1_, Ω_2_, Ω_3_, ⋯, Ω_*N*_} be a finite population of *N* units. Let *y*_*i*_ and (*x*_*i*_, *z*_*i*_) be the values of the study variable (*y*) and the auxiliary variable (*x*, *z*), respectively, for *i* = 1, 2, ⋯, *N*. Assume that *x*_*i*_ has high positive and *z*_*i*_ has low positive correlation, respectively, with the study variable (*y*_*i*_). So, *x*_*i*_ is used at the estimation stage and *z*_*i*_ is used at the sample selection stage from population. Let a sample {(ℑ = ℑ_1_, ℑ_2_, ⋯, ℑ_*n*_)} of size *n* be selected using simple random sampling without replacement(SRSWOR) scheme. Assume that *n*_1*s*_ units respond at first call, report their responses *y*_*i*(1)_ and *n*_2*s*_ units do not respond at first call. Further, a sample of size $r_{1s}=\frac{n_{2s}}{k}$, where *k* > 1, is drawn from *n*_2*s*_ non-respondent group, report their responses *y*_*i*(2)_, belong to group *G*_1_ and *r*_2*s*_ = *r*_1*s*_(*k* − 1) are those, who refuse to report their response belong to group *G*_2_.

Thus, the sub-sampling estimate for population mean, is given by

$$\begin{array}{cc} {\bar{y}}^{*} & =\frac{1}{n}\{\sum_{i=1}^{n_{1s}}y_{i(1)}+\sum_{i=1}^{n_{2s}}y_{i(2)}\}\\ & =w_{1s}{\bar{y}}_{\left(1\right)}+w_{2s}{\bar{y}}_{\left(2\right)}, \end{array}$$ (1)

where $w_{1s}=\frac{n_{1s}}{n}$, $w_{2s}=\frac{n_{2s}}{n}$, ${\bar{y}}_{\left(1\right)}=\frac{1}{n_{1}}\sum_{i=1}^{n_{1}}y_{i}$, ${\bar{y}}_{\left(1\right)}=\frac{1}{r_{1}}\sum_{i=1}^{n_{2}}y_{i}$ and *r* be the respondents. The variance of ${\bar{y}}^{*}$ is given by

$$\begin{array}{c} V({\bar{y}}^{*})=\frac{\left(N-n\right)}{nN}{\bar{Y}}^{2}C_{y}^{2}+\frac{1}{n}W_{2}(k-1){\bar{Y}}_{2}^{2}C_{y_{\left(2\right)}}^{2}, \end{array}$$ (2)

where $\bar{Y}=\frac{1}{N}\sum_{i=1}^{N}y_{i}$, $S_{y}^{2}=\frac{1}{N-1}\sum_{i=1}^{N}{\left(y_{i}-\bar{Y}\right)}^{2}$, $C_{y}^{2}=\frac{S_{y}^{2}}{{\bar{Y}}^{2}}$,${\bar{Y}}_{2}=\frac{1}{N_{2}}\sum_{i=1}^{N_{2}}y_{i}$, $S_{y(2)}^{2}=\frac{1}{N_{2}-1}\sum_{i=1}^{N_{2}}{\left(y_{i}-{\bar{Y}}_{2}\right)}^{2}$, $C_{y(2)}^{2}=\frac{S_{y(2)}^{2}}{{\bar{Y}}_{2}^{2}}$, and $W_{2}=\frac{N_{2}}{N}$.

### 1.2 Selection of sample with PPS sampling

In PPS sampling scheme, the selection of units in the sample is carried with probability proportional to a given measure of size, where the size is measured by the available suitable auxiliary information. Let *u*_*i*_ = *y*_*i*_/(*Nπ*_*i*_) and *v*_*i*_ = *x*_*i*_/(*Nπ*_*i*_), where $\pi_{i}=z_{i}/\sum_{i=1}^{N}z_{i}$ and also let $\bar{u}=\frac{1}{n}\sum_{i=1}^{n}u_{i}$ and $\bar{v}=\frac{1}{n}\sum_{i=1}^{n}v_{i}$ be the unbiased estimators of population means and their variances are $V(\bar{u})=\sum_{i=1}^{n}p_{i}{\left(u_{i}-\bar{u}\right)}^{2}$ and $V(\bar{v})=\sum_{i=1}^{n}p_{i}{\left(v_{i}-\bar{v}\right)}^{2}$, respectively, where $p_{i}=z_{i}/\sum_{i=1}^{n}z_{i}$. It is also assumed that the average value of *u*_*i*_ is approximately equal to average value of *y*_*i*_.

Let a sample {*s*_*i*_ = (*s*_1_, *s*_2_, *s*_3_, ⋯, *s*_*n*_)} of size *n* be selected using PPS with replacement sampling scheme. Assume that *n*_1_ units respond at first call, report their responses *u*_*i*(1)_ = *y*_*i*(1)_/(*N*_1_ *π*_1*i*_), where $\pi_{1i}=z_{i}/\sum_{i=1}^{N_{1}}z_{i}$ and *n*_2_ units do not respond at first call. Further, a sample of size, $r_{1}=\frac{n_{2}}{k}$, is drawn from *n*_2_ non-respondent group, report their responses *u*_*i*(2)_ = *y*_*i*(2)_/(*N*_2_*π*_2*i*_), where $\pi_{2i}=z_{i}/\sum_{i=1}^{N_{2}}z_{i}$, belongs to group *G*_1_ and *r*_2_ = *r*_1_(*k* − 1) is those, who refuse to report their responses belong to group *G*_2_. Thus, the Hansen-Hurwitz estimator under PPS sampling scheme can be modified as:

$$\begin{array}{cc} {\bar{u}}^{*} & =\frac{1}{n}\{\sum_{i=1}^{n_{1}}u_{i(1)}+\sum_{i=1}^{n_{2}}u_{i(2)}\}\\ & =\frac{1}{n}\{n_{1}{\bar{u}}_{\left(1\right)}+n_{2}{\bar{u}}_{\left(2\right)}\}\\ & =w_{1}{\bar{u}}_{\left(1\right)}+w_{2}{\bar{u}}_{\left(2\right)}, \end{array}$$ (3)

where *n*_1_ and *r*_1_ are the PPS respondent units at first and second calls, respectively. The variance of ${\bar{u}}^{*}$ is given by

$$\begin{array}{c} V({\bar{u}}^{*})=\frac{\left(N-n\right)}{nN}{\bar{Y}}^{2}C_{u}^{2}+\frac{1}{n}W_{2}(k-1){\bar{Y}}_{2}^{2}C_{u_{\left(2\right)}}^{2}, \end{array}$$ (4)

where $S_{u}^{2}=\sum_{i=1}^{N}\pi_{i}{\left(u_{i}-\bar{Y}\right)}^{2}$, $S_{u_{\left(2\right)}}^{2}=\sum_{i=1}^{N_{2}}\pi_{2i}{\left(u_{i(2)}-{\bar{Y}}_{2}\right)}^{2}$, $C_{u}^{2}=\frac{S_{u}^{2}}{{\bar{Y}}^{2}}$, and $C_{u_{\left(2\right)}}^{2}=\frac{S_{u(2)}^{2}}{{\bar{Y}}_{2}^{2}}$.

### 1.3 Statement of the problem

When variables of interest are sensitive or embarrassing in nature, then respondents are reluctant to report their true responses or may refuse to respond. Several statistical models are available in literature to protect the confidentiality and privacy of interviewee by hiding their identities, which are helpful to reduce the non-response bias. A pioneer idea of randomized response technique (RRT) was described by [2] to handle the high rate of refusals due to sensitive nature of questions. Commonly, these refusals have been occurred during the analysis of demographic and economic variables, respectively, etc. Interest readers may be referred to read [3–9], and many others. [10, 11] use the randomized response models (RRMs) for obtaining the true status of interviewee on second attempt. The proposed estimators by these researchers can perform better as compared to traditional ones.

The aim of this investigation is to study the missing complete at random (MCAR) values at second call, when the interviewees are reluctant to use RRMs. For the non-respondents of first call, different additive, multiplicative and subtractive models, respectively, might be utilized to create the feeling among respondents that their privacy is secured beside their truthful response.

For creating privacy protection felling among non-respondents of first call, we consider to modify linear randomized response model proposed by [12]. From the *n*_2_ non-respondents of first call, the scrambled response is obtained using the [12] model.

#### 1.3.1 Privacy protection at second call

Let the *i*^*th*^ respondent draw two cards i.e *S*_1*i*_ and *S*_2*i*_ from two independent decks of cards, say *D*_1_ and *D*_2_, respectively, which are un-correlated with *y*. At the second call, the *i*^*th*^ respondent can report the scrambled response as follows:

$$\begin{array}{c} u_{i(2)}^{{}^{\prime }}=S_{1i}u_{i(2)}+S_{2i} \end{array}$$ (5)

Let *E*_3_ and *V*_3_ be, respectively, the expected value and variance over the scrambled device. We assume that *E*_3_(*S*_1*i*_) = *θ*_1_, *E*_3_(*S*_2*i*_) = *θ*_2_, $V_{3}\left(S_{1i}\right)=\sigma_{1}^{2}$ and $V_{3}(S_{2i})=\sigma_{2}^{2}$ with $E_{3}(u_{i(2)}^{{}^{\prime }})=\theta_{1}u_{i(2)}+\theta_{2}$ and $V_{3}(u_{i(2)}^{{}^{\prime }})=\sigma_{1}^{2}u_{i(2)}^{2}+\sigma_{2}^{2}$. Also let ${\hat{u}}_{i(2)}$ be the suitable transformation of randomized response for the *i*^*th*^ unit whose expectation under (5) model coincides with the true response *y*_*i*_, as:

$$\begin{array}{c} {\hat{u}}_{i(2)}=\frac{\theta_{2}}{\theta_{1}}(\frac{u_{i(2)}^{{}^{\prime }}}{\theta_{2}}-1) \end{array}$$ (6)

with

$$\begin{array}{c} V_{3}({\hat{u}}_{i(2)})=C_{\theta_{1}}^{2}{\hat{u}}_{i}^{2}+{\left(\frac{\theta_{2}}{\theta_{1}}\right)}^{2}C_{\theta_{2}}^{2}, \end{array}$$ (7)

where $C_{\theta_{1}}^{2}=\frac{\sigma_{1}^{2}}{\theta_{1}^{2}}$ and $C_{\theta_{2}}^{2}=\frac{\sigma_{2}^{2}}{\theta_{2}^{2}}$.

At the second call, out of *n*_2_ non-respondent of first call, only *r*_1_ interviewees can give their scrambling responses and remaining *r*_2_ units cannot give their true or scrambled responses. Let ${\hat{\bar{u}}}_{\left(r_{1}\right)}=\frac{1}{r_{1}}\sum_{i=1}^{r_{1}}{\hat{u}}_{i(r_{1})}$ be the sample mean of respondent class at second attempt.

## 2 Modifying existing literature

In this section, we modify the exiting literature as per the statement of the problem. The most commonly used imputation procedures are discussed in Subsection 2.1, 2.2, and 2.3.

### 2.1 Mean estimator

In this section, our focus is to impute the missing *r*_2_ values by using conventional method of imputation. The missing structure is defined as follows:

$$\begin{array}{c} \Delta_{i}=\{\begin{array}{cc} {\hat{u}}_{i(r_{1})} & \text{if}\;\text{the}\;i^{th}\;\text{respondent}\;\text{report}\;\text{their}\;\text{scrambled}\;\text{response}\;\\ {\hat{\bar{u}}}_{\left(r_{1}\right)} & \text{otherwise} \end{array} \end{array}$$ (8)

Hence, the whole population is divided in Ω_(1)_ and Ω_(2)_ strata having *N*_1_ and *N*_2_ units, respectively. Furthermore, Ω_(2)_ is divided into two groups *G*_1_ and *G*_2_ of size *R*_1_ and *R*_2_ units, respectively, when *N*_1_, *N*_2_, *R*_1_ and *R*_2_ are known in advance. For the case of scrambled responses at second call, the point Hansen-Hurwitz estimator for population mean $\left(\bar{Y}\right)$ can be modified as:

$$\begin{array}{cc} {\hat{\bar{u}}}^{*} & =\frac{1}{n}\sum_{i=1}^{n}\Delta_{i}\\ & =\frac{1}{n}\{\sum_{i=1}^{n_{1}}\Delta_{i}+\sum_{i=1}^{n_{2}}\Delta_{i}\}\\ & =\frac{1}{n}\{n_{1}{\bar{u}}_{\left(1\right)}+\sum_{i=1}^{r_{1}}u_{i(r_{1})}+\sum_{i=1}^{r_{2}}{\hat{\bar{u}}}_{\left(r_{1}\right)}\}\\ & =\frac{1}{n}\{n_{1}{\bar{u}}_{1}+r_{1}{\hat{\bar{u}}}_{\left(r_{1}\right)}+\left(n_{2}-r_{1}\right){\hat{\bar{u}}}_{\left(r_{1}\right)}\}\\ & =w_{1}{\bar{u}}_{\left(1\right)}+w_{2}{\hat{\bar{u}}}_{\left(r_{1}\right)} \end{array}$$ (9)

So, we have the following Lemmas.

**Lemma 2.1** The variance of ${\hat{\bar{u}}}_{\left(r_{1}\right)}$, is given by

$$\begin{array}{c} V\{{\hat{\bar{u}}}_{\left(r_{1}\right)}\}=\frac{1}{N_{2}r_{1}}\sum_{i=1}^{N_{2}}\phi_{i}+(\frac{1}{r_{1}}-\frac{1}{N_{2}}){\bar{Y}}_{2}^{2}C_{u_{\left(2\right)}}^{2}. \end{array}$$ (10)

*Proof*. **Proof**: Let *E*_*j*_ and *V*_*j*_, *j* = (1, 2) be the expected values and variances for given *n*_2_ and *r*_1_, respectively. Then, by the definition of variance, we have

$$\begin{array}{cc} V\{{\hat{\bar{u}}}_{\left(r_{1}\right)}\} & =E_{1}E_{2}V_{3}\{{\hat{\bar{u}}}_{\left(r_{1}\right)}\}+E_{1}V_{2}E_{3}\{{\hat{\bar{u}}}_{\left(r_{1}\right)}\}+V_{1}E_{2}E_{3}\{{\hat{\bar{u}}}_{\left(r_{1}\right)}\}\\ & =E_{1}E_{2}V_{3}\{\frac{1}{r_{1}}\sum_{i=1}^{r_{1}}{\hat{u}}_{i}\}+E_{1}V_{2}E_{3}\{\frac{1}{r_{1}}\sum_{i=1}^{r_{1}}{\hat{u}}_{i}\}+V_{1}E_{2}E_{3}\{\frac{1}{r_{1}}\sum_{i=1}^{r_{1}}{\hat{u}}_{i}\}\\ & =E_{1}E_{2}\{\frac{1}{r_{1}^{2}}\sum_{i=1}^{r}V_{3}({\hat{u}}_{i})\}+E_{1}V_{2}\{\frac{1}{r_{1}}\sum_{i=1}^{r_{1}}E_{3}({\hat{u}}_{i})\}+V_{1}E_{2}\{\frac{1}{r_{1}}\sum_{i=1}^{r}E_{3}({\hat{u}}_{i})\}\\ & =E_{1}E_{2}[\frac{1}{r_{1}^{2}}\sum_{i=1}^{r_{1}}\{C_{\theta_{1}}^{2}{\hat{u}}_{i}^{2}+{\left(\frac{\theta_{2}}{\theta_{1}}\right)}^{2}C_{\theta_{2}}^{2}\}]+E_{1}V_{2}\{\frac{1}{r_{1}}\sum_{i=1}^{r}u_{i(2)}\}\\ & \;+V_{1}E_{2}\{\frac{1}{r_{1}}\sum_{i=1}^{r_{1}}u_{i(2)}\}\\ & =E_{1}[\frac{1}{n_{2}r_{1}}\sum_{i=1}^{n_{2}}\{C_{\theta_{1}}^{2}(\frac{N_{2}-1}{N_{2}}S_{u_{\left(2\right)}}^{2}+{\bar{Y}}_{2}^{2})+{\left(\frac{\theta_{2}}{\theta_{1}}\right)}^{2}C_{\theta_{2}}^{2}\}]\\ & \;+E_{1}(\frac{1}{r_{1}}-\frac{1}{n_{2}})s_{u_{\left(2\right)}}^{2}+V_{1}\{\frac{1}{n_{2}}\sum_{i=1}^{n_{2}}u_{\left(2\right)}\}\\ & =\frac{1}{N_{2}r_{1}}\sum_{i=1}^{N_{2}}\{{\bar{Y}}_{2}^{2}C_{\theta_{1}}^{2}(1+\frac{N_{2}-1}{N_{2}}C_{u_{\left(2\right)}}^{2})+{\left(\frac{\theta_{2}}{\theta_{1}}\right)}^{2}C_{\theta_{2}}^{2}\}+(\frac{1}{r_{1}}-\frac{1}{n_{2}}){\bar{Y}}_{2}^{2}C_{u_{\left(2\right)}}^{2}\\ & \;+(\frac{1}{n_{2}}-\frac{1}{N_{2}}){\bar{Y}}_{2}^{2}C_{u_{\left(2\right)}}^{2}.\\ & =\frac{1}{N_{2}r_{1}}\sum_{i=1}^{N_{2}}\{{\bar{Y}}_{2}^{2}C_{\theta_{1}}^{2}(1+\frac{N_{2}-1}{N_{2}}C_{u_{\left(2\right)}}^{2})+{\left(\frac{\theta_{2}}{\theta_{1}}\right)}^{2}C_{\theta_{2}}^{2}\}+(\frac{1}{r_{1}}-\frac{1}{N_{2}}){\bar{Y}}_{2}^{2}C_{u_{\left(2\right)}}^{2}\\ & =\frac{1}{N_{2}r_{1}}\sum_{i=1}^{N_{2}}\phi_{i}+(\frac{1}{r_{1}}-\frac{1}{N_{2}}){\bar{Y}}_{2}^{2}C_{u_{\left(2\right)}}^{2}. \end{array}$$ (11)

**Corollary 2.1.1**. It is important to note that $V\{{\hat{\bar{u}}}_{\left(r_{1}\right)}\}$ requires the second moment (*μ*_2*u*_) of *y*, which is generally unknown. [13] suggested two possible ways to acquire *μ*_2*u*_: (i) guess it from the prior information or pilot survey and (ii) obtain the sample estimate to derive the information about *μ*_2*u*_ by keeping in mind the sensitive nature of *u*_*i*_.

**Lemma 2.2**. The variance of ${\hat{\bar{u}}}^{*}$, is given by

$$\begin{array}{c} V({\hat{\bar{u}}}^{*})=\frac{k}{Nnr_{1}}\sum_{i=1}^{N_{2}}\phi_{i}+\frac{1}{n}W_{2}(k-1){\bar{Y}}_{2}^{2}C_{u_{\left(2\right)}}^{2}+\frac{\left(N-n\right)}{nN}{\bar{Y}}^{2}C_{u}^{2}. \end{array}$$ (12)

*Proof*. **Proof** Let *E*_*m*_ and *V*_*m*_, *m* = (4, 5) be the expected values and variances for given *N*_1_ and *N*_2_, respectively. By definition, we have

$$\begin{array}{ccc} V({\hat{\bar{u}}}^{*}) & = & E_{4}V_{5}\{{\hat{\bar{u}}}^{*}|n_{1},n_{2}\}+V_{4}E_{5}\{{\hat{\bar{u}}}^{*}|n_{1},n_{2}\}\\ & = & E_{4}V_{5}\{\frac{w_{1}{\bar{u}}_{\left(1\right)}+w_{2}{\hat{\bar{u}}}_{\left(r_{1}\right)}}{n}|n_{1},n_{2}\}+V_{4}E_{5}\{\frac{w_{1}{\bar{u}}_{\left(1\right)}+w_{2}{\hat{\bar{u}}}_{\left(r_{1}\right)}}{n}|n_{1},n_{2}\}\\ & = & E_{4}\{\frac{1}{N_{2}r_{1}}\sum_{i=1}^{N_{2}}\phi_{i}+\frac{n_{2}^{2}}{n^{2}}(\frac{1}{r_{1}}-\frac{1}{n_{2}})s_{y_{\left(2\right)}}^{2}\}+V_{4}\{{\bar{u}}_{\left(n\right)}\}\\ & = & E_{4}\{\frac{1}{N_{2}r_{1}}\sum_{i=1}^{N_{2}}\phi_{i}+\frac{1}{n}(\frac{n_{2}}{r_{1}}-1)\frac{n_{2}}{n}s_{u_{\left(2\right)}}^{2}\}+V_{4}\{{\bar{u}}_{\left(n\right)}\}\\ & = & \frac{k}{Nnr_{1}}\sum_{i=1}^{N_{2}}\phi_{i}+\frac{1}{n}W_{2}(k-1){\bar{Y}}_{2}^{2}C_{u_{\left(2\right)}}^{2}+\frac{\left(N-n\right)}{nN}{\bar{Y}}^{2}C_{u}^{2}. \end{array}$$ (13)

By ignoring correction factor $\left(1-\frac{n}{N}\right)$ for the ease of computation, then we have

$$\begin{array}{c} V({\hat{\bar{u}}}^{*})=\frac{k}{Nnr_{1}}\sum_{i=1}^{N_{2}}\phi_{i}+\frac{1}{n}W_{2}(k-1){\bar{Y}}_{2}^{2}C_{u_{\left(2\right)}}^{2}+\frac{1}{n}{\bar{Y}}^{2}C_{u}^{2}. \end{array}$$ (14)

**Corollary 2.2.1**. From (4) and (14), we see that the variance of modified estimator is higher than Hansen-Hurwitz estimator. It means that ${\hat{\bar{u}}}^{*}$ is less efficient than ${\bar{u}}^{*}$.

The objective of our study is to increase the truth and confidence among interviewees that their privacy is secure beside their true answers. Moreover, the non-response at first call might be occurred due to non-availability or inability to provide the required information. Therefore, at the second call, it may happen that those people are willing to report their responses directly, even the sensitive characteristics are investigated. For this purpose, the randomization in stages should be re-expounded as an optional randomized response (ORR) procedure, which permits the respondents to divulging the direct or true response without using RRT, is given by

$$\begin{array}{c} {\hat{u}}_{i}=t_{i}u_{i}+\left(1-t_{i}\right)u_{i(1)}, \end{array}$$ (15)

where

$$\begin{array}{c} t_{i}=\{\begin{array}{cc} 1 & \text{if}\;\text{the}\;i^{\text{th}}\;\text{respondent}\;\text{report}\;\text{their}\;\text{direct}\;\text{response}\\ 0 & \text{otherwise} \end{array} \end{array}$$

It is easy to show that the unbiased estimator for $\bar{Y}$ is derived by replacing (15) in (9) and its variance becomes (1 − *t*_*i*_)*ϕ*_*i*_ instead of *ϕ*_*i*_, in (14). Furthermore, ORR reduces the variance and privacy at various values of *t*_*i*_ for the non-respondents at first call.

### 2.2 Ratio estimator

Initially, [14] takes into account the utility of auxiliary information at estimation stage by defining the ratio estimator for population. The traditional ratio estimator can be modified for the imputation of missing scrambled responses at second call, as:

$$\begin{array}{c} {△}_{i(c)}=\{\begin{array}{cc} {\hat{u}}_{i(r_{1})} & \text{if}\;i\;\;\in \;G_{1}\\ \frac{1}{1-f_{1}}({\hat{\bar{u}}}_{\left(r_{1}\right)}\frac{{\bar{X}}_{2}}{{\bar{v}}_{\left(r_{1}\right)}}-f_{1}{\hat{\bar{u}}}_{\left(r_{1}\right)}) & \text{if}\;i\;\;\in \;G_{2} \end{array}, \end{array}$$ (16)

where $f_{1}=\frac{r_{1}}{n_{2}}$, ${\bar{v}}_{r_{1}}=\frac{1}{r_{1}}\sum_{i=1}^{r_{1}}\frac{x_{i}}{p_{2i}}$, and ${\bar{X}}_{2}=\frac{1}{N_{2}}\sum_{i=1}^{N_{2}}x_{i}$.

The point estimator for sub-population (Ω_(2)_), is given by

$$\begin{array}{cc} {\hat{\bar{u}}}_{\left(c\right)} & =\frac{1}{n_{2}}\sum_{i=1}^{n_{2}}\Delta_{i(c)}\\ & =\frac{1}{n_{2}}\{\sum_{i=1}^{r_{1}}\Delta_{i(c)}+\sum_{i=1}^{r_{2}}\Delta_{i(c)}\}\\ & =\frac{1}{n_{2}}\{\sum_{i=1}^{r_{1}}y_{i(r_{1})}+\frac{1}{1-f_{1}}\sum_{i=1}^{r_{2}}({\hat{\bar{u}}}_{\left(r_{1}\right)}\frac{{\bar{X}}_{2}}{{\bar{v}}_{r_{1}}}-f_{1}{\hat{\bar{u}}}_{\left(r_{1}\right)})\}\\ & =\frac{1}{n_{2}}\{r_{1}{\hat{\bar{u}}}_{\left(r_{1}\right)}+n_{2}{\hat{\bar{u}}}_{\left(r_{1}\right)}\frac{{\bar{X}}_{2}}{{\bar{v}}_{\left(r_{1}\right)}}-r_{1}{\hat{\bar{u}}}_{\left(r_{1}\right)}\}\\ & ={\hat{\bar{u}}}_{\left(r_{1}\right)}\frac{{\bar{X}}_{2}}{{\bar{v}}_{\left(r_{1}\right)}} \end{array}$$ (17)

The Hansen-Hurwitz ratio estimator for population mean $\left(\bar{Y}\right)$, is given by

$$\begin{array}{c} {\hat{\bar{u}}}_{\left(c\right)}^{*}=w_{1}{\bar{u}}_{\left(1\right)}+w_{2}{\hat{\bar{u}}}_{\left(c\right)} \end{array}$$ (18)

The variance of modified ratio estimator is given by

$$\begin{array}{ccc} V({\hat{\bar{u}}}_{\left(c\right)}^{*}) & = & E_{4}V_{5}\{{\hat{\bar{u}}}_{\left(c\right)}^{*}|n_{1},n_{2}\}+V_{4}E_{5}\{{\hat{\bar{u}}}_{\left(c\right)}^{*}|n_{1},n_{2}\}\\ & = & E_{4}V_{5}\{\frac{w_{1}{\bar{u}}_{\left(1\right)}+w_{2}{\hat{\bar{u}}}_{\left(c\right)}}{n}|n_{1},n_{2}\}+V_{4}E_{5}\{\frac{w_{1}{\bar{u}}_{\left(1\right)}+w_{2}{\hat{\bar{u}}}_{\left(c\right)}}{n}|n_{1},n_{2}\}\\ & = & E_{4}\{\frac{1}{N_{2}r_{1}}\sum_{i=1}^{N_{2}}\phi_{i}+\frac{n_{2}^{2}}{n^{2}}(\frac{1}{r_{1}}-\frac{1}{n_{2}})s_{r_{\left(2\right)}}^{2}\}+V_{4}\{{\bar{u}}_{\left(n\right)}\}\\ & = & E_{4}\{\frac{1}{N_{2}r_{1}}\sum_{i=1}^{N_{2}}\phi_{i}+\frac{1}{n}(\frac{n_{2}}{r_{1}}-1)\frac{n_{2}}{n}s_{r_{\left(2\right)}}^{2}\}+V_{4}\{{\bar{u}}_{\left(n\right)}\}\\ & = & \frac{k}{Nnr_{1}}\sum_{i=1}^{N_{2}}\phi_{i}+\frac{1}{n}W_{2}(k-1){\bar{Y}}_{2}^{2}C_{r_{\left(2\right)}}^{2}+\frac{1}{n}{\bar{Y}}_{2}^{2}C_{y_{}}^{2}, \end{array}$$ (19)

where $C_{r_{\left(2\right)}}^{2}=C_{u_{\left(2\right)}}^{2}+C_{v_{\left(2\right)}}^{2}-2\rho_{uv}C_{u_{\left(2\right)}}C_{v_{\left(2\right)}}$,

$$\begin{array}{cc} C_{u_{\left(2\right)}}^{2} & =\frac{S_{u_{\left(2\right)}}^{2}}{{\bar{Y}}_{2}},\;C_{v_{\left(2\right)}}^{2}=\frac{S_{v_{\left(2\right)}}^{2}}{{\bar{X}}_{2}},\rho_{uv}=\frac{S_{uv_{\left(2\right)}}}{S_{u_{\left(2\right)}}S_{v_{\left(2\right)}}},S_{u_{\left(2\right)}}^{2}=\frac{1}{N_{2}-1}\sum_{i=1}^{N_{2}}\pi_{2i}{\left(u_{i}-{\bar{Y}}_{2}\right)}^{2},\\ S_{v_{\left(2\right)}}^{2} & =\frac{1}{N_{2}-1}\sum_{i=1}^{N_{2}}\pi_{2i}{\left(v_{i}-{\bar{X}}_{2}\right)}^{2},S_{uv_{\left(2\right)}}=\frac{1}{N_{2}-1}\sum_{i=1}^{N_{2}}\pi_{2i}(u_{i}-{\bar{Y}}_{2})(v_{i}-{\bar{X}}_{2}). \end{array}$$

### 2.3 Difference estimator

Now, we consider the difference estimator for explaining missing structure of scrambled responses, as:

$$\begin{array}{c} {△}_{i(d)}=\{\begin{array}{cc} {\hat{u}}_{i(r_{1})} & \text{if}\;i\;\;\in \;G_{1}\\ \frac{1}{1-f_{1}}\{{\hat{\bar{u}}}_{\left(r_{1}\right)}+d({\bar{X}}_{2}-{\bar{v}}_{\left(r_{1}\right)})-f_{1}{\hat{\bar{u}}}_{\left(r_{1}\right)}\} & \text{if}\;i\;\;\in \;G_{2} \end{array}, \end{array}$$ (20)

where *d* is an unknown constant.

The point estimator for sub-population mean (Ω), is given by

$$\begin{array}{cc} {\hat{\bar{u}}}_{\left(d\right)} & =\frac{1}{n_{2}}\sum_{i=1}^{n_{2}}\Delta_{i(d)}\\ & =\frac{1}{n_{2}}\{\sum_{i=1}^{r_{1}}\Delta_{i(d)}+\sum_{i=1}^{r_{2}}\Delta_{i(d)}\}\\ & =\frac{1}{n_{2}}\{\sum_{i=1}^{r_{1}}u_{i(r_{1})}+\frac{1}{1-f_{1}}\sum_{i=1}^{r_{2}}({\hat{\bar{u}}}_{\left(r_{1}\right)}+d({\bar{X}}_{2}-{\bar{v}}_{\left(r_{1}\right)})-f_{1}{\hat{\bar{u}}}_{\left(r_{1}\right)})\}\\ & =\frac{1}{n_{2}}\{r_{1}{\hat{\bar{u}}}_{\left(r_{1}\right)}+n_{2}\{{\hat{\bar{u}}}_{\left(r_{1}\right)}+d({\bar{X}}_{2}-{\bar{v}}_{\left(r_{1}\right)})\}-r_{1}{\hat{\bar{u}}}_{\left(r_{1}\right)}\}\\ & ={\hat{\bar{u}}}_{\left(r_{1}\right)}+d({\bar{X}}_{2}-{\bar{v}}_{\left(r_{1}\right)}) \end{array}$$ (21)

The combined version of modified Hansen-Hurwitz estimator is given by

$$\begin{array}{c} {\hat{\bar{u}}}_{\left(d\right)}^{*}=w_{1}{\bar{u}}_{\left(1\right)}+w_{2}{\hat{\bar{u}}}_{\left(d\right)} \end{array}$$ (22)

The variance of ${\hat{\bar{u}}}_{\left(d\right)}^{*}$ estimator, is stated as

$$\begin{array}{ccc} V({\hat{\bar{u}}}_{\left(d\right)}^{*}) & = & E_{4}V_{5}\{{\hat{\bar{u}}}_{\left(d\right)}^{*}|n_{1},n_{2}\}+V_{4}E_{5}\{{\hat{\bar{u}}}_{\left(d\right)}^{*}|n_{1},n_{2}\}\\ & = & E_{4}V_{5}\{\frac{w_{1}{\bar{u}}_{\left(1\right)}+w_{2}{\hat{\bar{u}}}_{\left(d\right)}}{n}|n_{1},n_{2}\}+V_{4}E_{5}\{\frac{w_{1}{\bar{u}}_{\left(1\right)}+w_{2}{\hat{\bar{u}}}_{\left(d\right)}}{n}|n_{1},n_{2}\}\\ & = & E_{4}\{\frac{1}{N_{2}r_{1}}\sum_{i=1}^{N_{2}}\phi_{i}+\frac{n_{2}^{2}}{n^{2}}(\frac{1}{r_{1}}-\frac{1}{n_{2}})s_{d_{\left(2\right)}}^{2}\}+V_{4}\{{\bar{u}}_{\left(n\right)}\}\\ & = & E_{4}\{\frac{1}{N_{2}r_{1}}\sum_{i=1}^{N_{2}}\phi_{i}+\frac{1}{n}(\frac{n_{2}}{r_{1}}-1)\frac{n_{2}}{n}s_{d_{\left(2\right)}}^{2}\}+V_{4}\{{\bar{u}}_{\left(n\right)}\}\\ & = & \frac{k}{Nnr_{1}}\sum_{i=1}^{N_{2}}\phi_{i}+\frac{1}{n}W_{2}(k-1)C_{d_{\left(2\right)}}^{2}+\frac{1}{n}{\bar{Y}}_{2}^{2}C_{y_{}}^{2}, \end{array}$$ (23)

where $C_{d_{\left(2\right)}}^{2}={\bar{Y}}_{2}^{2}C_{u_{\left(2\right)}}^{2}+d^{2}{\bar{X}}_{2}^{2}C_{v_{\left(2\right)}}^{2}-2k{\bar{Y}}_{2}{\bar{X}}_{2}\rho_{uv_{\left(2\right)}}C_{u_{\left(2\right)}}C_{v_{\left(2\right)}}$.

When $d_{\text{opt.}}=\frac{{\bar{Y}}_{2}}{{\bar{X}}_{2}}\frac{C_{u_{\left(2\right)}}}{C_{v_{\left(2\right)}}}\rho_{uv_{\left(2\right)}}$, variance of ${\hat{\bar{u}}}_{\left(d\right)}^{*}$ reduces to

$$\begin{array}{c} V({\hat{\bar{u}}}_{\left(d\right)}^{*})≅\frac{1}{Nnr_{1}}\sum_{i=1}^{N_{2}}\phi_{i}+\frac{1}{n}W_{2}\left(k-1\right){\bar{Y}}_{2}^{2}C_{d_{\left(2\right)}}^{2*}+\frac{1}{n}{\bar{Y}}^{2}C_{u_{}}^{2}, \end{array}$$ (24)

where $C_{d_{\left(2\right)}}^{2*}=C_{u}^{2}(1-\rho_{uv_{\left(2\right)}}^{2})$.

The problem of estimating the population parameters by using higher order moments of the auxiliary variable was considered by [15–17]. Later on [18–20] among others, also contemplate the known higher order moments of the auxiliary variable for estimation of finite population parameters. In the theory of survey sampling, it is well established result that the use of higher order moments of the auxiliary variable plays a pivotal role in estimating the finite population mean of the study variable. This literature inspired the researchers to impute the missing values at second call by using known covariance between the study variable and the auxiliary variable.

## 3 Proposed imputation procedure

Initially, [21] improves the conventional mean estimator by using a tuning constant (*α*_(*s*)_), in the case of missing values, as:

$$\begin{array}{c} {△}_{i(s)}=\{\begin{array}{cc} \alpha_{\left(s\right)}{\hat{u}}_{i(r_{1})} & \text{if}\;i\;\;\in \;G_{1}\\ \alpha_{\left(s\right)}{\hat{\bar{u}}}_{\left(r_{1}\right)} & \text{if}\;i\;\;\in \;G_{2} \end{array}, \end{array}$$ (25)

which leads to Searls’s type estimator for ${\bar{u}}_{\left(2\right)}$ is given by

$$\begin{array}{c} {\bar{y}}_{\left(s\right)}=\alpha_{\left(s\right)}{\hat{\bar{u}}}_{\left(r_{1}\right)}. \end{array}$$ (26)

Although Searls’s approach uses the known coefficient of variation to increase the efficiency of the estimation procedure. The optimum value of *α*_(*s*)_ depends on $C_{u_{\left(2\right)}}$, $C_{v_{\left(2\right)}}$ and $\rho_{uv_{\left(2\right)}}$, which are stable quantities. The stability of these constant has been explored by numerous researchers like [22–24], etc. Therefor, the present investigation is a significant search of optimum imputation method by using the co-variance between the study and auxiliary variable. The imputation of item non-response is given by

$$\begin{array}{c} {△}_{i(p)}=\{\begin{array}{cc} \alpha_{1}{\hat{u}}_{i(r_{1})} & \text{if}\;i\;\;\in \;G_{1}\\ \alpha_{1}{\hat{\bar{u}}}_{\left(r_{1}\right)}+\frac{\alpha_{2}}{1-f_{1}}({\bar{X}}_{2}-{\bar{v}}_{\left(r_{1}\right)})+\frac{\alpha_{3}}{1-f_{1}}(S_{uv_{\left(2\right)}}-s_{uv_{\left(r_{1}\right)}}) & \text{if}\;i\;\;\in \;G_{2} \end{array}, \end{array}$$ (27)

where *α*_1_, *α*_2_, and *α*_3_ are suitable chosen constants and are determined by minimizing the resultant mean square error. The point estimator for population mean, is defined as:

$$\begin{array}{cc} {\hat{\bar{u}}}_{\left(p\right)} & =\frac{1}{n_{2}}\sum_{i=1}^{n_{2}}\Delta_{i(p)}\\ & =\frac{1}{n_{2}}\{\sum_{i=1}^{r_{1}}\Delta_{i(p)}+\sum_{i=1}^{r_{2}}\Delta_{i(p)}\}\\ & =\frac{1}{n_{2}}\{\sum_{i=1}^{r_{1}}\alpha_{1}u_{i(r_{1})}+\sum_{i=1}^{r_{2}}(\alpha_{1}{\hat{\bar{u}}}_{\left(r_{1}\right)}+\frac{\alpha_{2}}{1-f_{1}}({\bar{X}}_{2}-{\bar{v}}_{\left(r_{1}\right)})+\frac{\alpha_{3}}{1-f_{1}}(S_{uv_{\left(2\right)}}-s_{uv_{\left(r_{1}\right)}}))\}\\ & =\frac{1}{n_{2}}\{\alpha_{1}r_{1}{\hat{\bar{u}}}_{\left(r_{1}\right)}+r_{2}(\alpha_{1}{\hat{\bar{u}}}_{\left(r_{1}\right)}+\frac{\alpha_{2}}{1-f_{1}}({\bar{X}}_{2}-{\bar{v}}_{\left(r_{1}\right)})+\frac{\alpha_{3}}{1-f_{1}}(S_{uv_{\left(2\right)}}-s_{uv_{\left(r_{1}\right)}}))\}\\ & =\alpha_{1}{\hat{\bar{u}}}_{\left(r_{1}\right)}+\alpha_{2}({\bar{X}}_{2}-{\bar{v}}_{\left(r_{1}\right)})+\alpha_{3}(S_{uv_{\left(2\right)}}-s_{uv_{\left(r_{1}\right)}}) \end{array}$$ (28)

The modified version of Hansen-Hurwitz difference estimator is given by

$$\begin{array}{cc} {\hat{\bar{u}}}_{\left(d\right)}^{*} & =w_{1}{\bar{u}}_{\left(1\right)}+w_{2}{\hat{\bar{u}}}_{\left(p\right)} \end{array}$$ (29)

The variance of ${\hat{\bar{u}}}_{\left(p\right)}$, is given by

$$\begin{array}{ccc} V({\hat{\bar{u}}}_{\left(p\right)}^{*}) & = & E_{4}V_{5}\{{\hat{\bar{u}}}_{\left(p\right)}^{*}|n_{1},n_{2}\}+V_{4}E_{5}\{{\hat{\bar{u}}}_{\left(p\right)}^{*}|n_{1},n_{2}\}\\ & = & E_{4}V_{5}\{\frac{w_{1}{\bar{u}}_{\left(1\right)}+w_{2}{\hat{\bar{u}}}_{\left(p\right)}}{n}|n_{1},n_{2}\}+V_{4}E_{5}\{\frac{w_{1}{\bar{u}}_{\left(1\right)}+w_{2}{\hat{\bar{u}}}_{\left(p\right)}}{n}|n_{1},n_{2}\}\\ & = & E_{4}\{\frac{1}{N_{2}r_{1}}\sum_{i=1}^{N_{2}}\phi_{i}+\frac{n_{2}^{2}}{n^{2}}(\frac{1}{r_{1}}-\frac{1}{n_{2}})s_{p_{\left(2\right)}}^{2}\}+V_{4}\{{\bar{u}}_{\left(n\right)}\}\\ & = & E_{4}\{\frac{1}{N_{2}r_{1}}\sum_{i=1}^{N_{2}}\phi_{i}+\frac{1}{n}(\frac{n_{2}}{r_{1}}-1)\frac{n_{2}}{n}s_{p_{\left(2\right)}}^{2}\}+V_{4}\{{\bar{u}}_{\left(n\right)}\}\\ & = & \frac{k}{Nnr_{1}}\sum_{i=1}^{N_{2}}\phi_{i}+\frac{1}{n}W_{2}(k-1)S_{p_{\left(2\right)}}^{2}+\frac{1}{n}{\bar{Y}}_{2}^{2}C_{y_{}}^{2}, \end{array}$$ (30)

where

$$\begin{array}{cc} S_{p_{\left(2\right)}}^{2} & =\alpha_{1}^{2}{\bar{Y}}_{2}^{2}C_{u_{\left(2\right)}}^{2}+\alpha_{2}^{2}{\bar{X}}^{2}C_{v_{\left(2\right)}}^{2*}+\alpha_{3}^{2}S_{uv_{\left(2\right)}}^{2}(\frac{\lambda_{22_{\left(2\right)}}}{\rho_{uv_{\left(2\right)}}^{}}-1)-2\;\alpha_{1}\alpha_{2}{\bar{Y}}_{2}{\bar{X}}_{2}\rho_{uv_{\left(2\right)}}C_{u_{\left(2\right)}}C_{v_{\left(2\right)}}\\ & -2\;\alpha_{1}\alpha_{3}{\bar{Y}}_{2}S_{uv_{\left(2\right)}}C_{u_{\left(2\right)}}\frac{\lambda_{21_{\left(2\right)}}}{\rho_{uv}}+2\;\alpha_{2}\;\alpha_{3}\;{\bar{Y}}_{2}S_{uv_{\left(2\right)}}C_{u_{\left(2\right)}}\frac{\lambda_{21_{\left(2\right)}}}{\rho_{uv_{\left(2\right)}}}+{\bar{Y}}_{2}^{2}{\left(\alpha_{1}-1\right)}^{2},\\ \lambda_{ab_{\left(2\right)}} & =\frac{\mu_{ab_{\left(2\right)}}}{\mu_{20_{\left(2\right)}}^{a/2}\mu_{02_{\left(2\right)}}^{b/2}},\;\mu_{ab_{\left(2\right)}}=\frac{1}{N_{2}-1}\sum_{i=1}^{N_{2}}\pi_{2i}{\left(u_{i_{\left(2\right)}}-{\bar{Y}}_{2}\right)}^{a}{\left(v_{i_{\left(2\right)}}-{\bar{X}}_{2}\right)}^{b}. \end{array}$$

The optimum values of *α*_*j*_, *j* = (1, 2, 3) are obtained, respectively, by minimizing (30), as follows:

$$\begin{array}{cc} \alpha_{1\left(\text{opt.}\right)} & =\frac{1}{1+\chi_{\left(2\right)}^{2}},\\ \alpha_{2\left(\text{opt.}\right)} & =\frac{\bar{Y_{2}}C_{u_{\left(2\right)}}\{\lambda_{21_{\left(2\right)}}\lambda_{12_{\left(2\right)}}-\varphi_{\left(2\right)}\rho_{uv_{\left(2\right)}}\}\alpha_{1\left(\text{opt.}\right)}}{\bar{X_{2}}C_{v_{\left(2\right)}}\{-\varphi_{\left(2\right)}+\lambda_{12_{\left(2\right)}}^{2}\}},\;\text{and}\;\\ \alpha_{3\left(\text{opt.}\right)} & =\frac{\bar{Y_{2}}C_{u_{\left(2\right)}}^{*}\rho_{uv}^{2}\{\lambda_{12_{\left(2\right)}}-\frac{\lambda_{21_{\left(2\right)}}}{\rho_{uv_{\left(2\right)}}^{2}}\}\alpha_{1\left(\text{opt.}\right)}}{S_{uv_{\left(2\right)}}\{-\varphi_{\left(2\right)}+\lambda_{12}^{2}\}}, \end{array}$$ (31)

where $\chi_{\left(2\right)}^{2}=C_{u_{\left(2\right)}}^{2*}(1-Q_{u.vs_{uv_{\left(2\right)}}}^{2})$, $\varphi_{\left(2\right)}=\rho_{uv_{\left(2\right)}}^{2}(\frac{\lambda_{22_{\left(2\right)}}}{\rho_{uv_{\left(2\right)}}^{2}}-1),$ and $Q_{u.vs_{uv_{\left(2\right)}}}^{2}=\{\lambda_{21_{\left(2\right)}}^{2}+\rho_{uv_{\left(2\right)}}^{2}\varphi_{\left(2\right)}-2\rho_{uv_{\left(2\right)}}^{*}\lambda_{21_{\left(2\right)}}\lambda_{12_{\left(2\right)}}\}{\left(\varphi_{\left(2\right)}-\lambda_{12_{\left(2\right)}}^{2}\right)}^{-1}$ is the coefficient of multiple determination of *u* on *v* and $s_{uv_{\left(2\right)}}$.

Substituting (31) in (30), the variance of ${\hat{\bar{u}}}_{\left(p\right)}$, is given by

$$\begin{array}{cc} V{\left({\hat{\bar{u}}}_{\left(p\right)}^{*}\right)}_{\text{min.}}≅ & \frac{1}{Nnr_{1}}\sum_{i=1}^{N_{2}}\phi_{i}+\frac{1}{n}W_{2}\left(k-1\right){\bar{Y}}_{2}^{2}C_{p_{\left(2\right)}}^{2*}+\frac{1}{n}\bar{Y}C_{u_{}}^{2}, \end{array}$$ (32)

where $C_{p_{\left(2\right)}}^{2*}=\chi_{\left(2\right)}^{2}{\left\{1+\chi_{\left(2\right)}^{2}\right\}}^{-1}$.

**Remark 1**. The second term in $V{\left({\hat{\bar{u}}}_{\left(p\right)}^{*}\right)}_{\text{min.}}$ is vanished, if *k* = 1. It happens when each non-respondent of first call is interviewed at second call.

## 4 Choice of sampling fractions

We shall deduce the optimum values of *k* and *n* that minimize the variance at specified cost. The cost function for the proposed model is based on following four components, as:

1. *C*_0_ = over head cost.
2. *C*_1_ = per unit cost for collecting the response by mail inquiry at first call.
3. *C*_2_ = the unit cost for obtaining the scrambled response from the non-respondent group of first call.
4. *C*_3_ = cost per unit for editing, processing or imputing the missing *r*_2_ values.

Thus, the cost function is given by

$$\begin{array}{c} C^{*}=nC_{0}+n_{1}C_{1}+r_{1}C_{2}+r_{2}C_{3} \end{array}$$ (33)

Note that C* is the total cost, thus it varies from sample to sample. So, we use the expected cost by applying the expectation on (33), we have

$$\begin{array}{cc} E(C^{*}) & =E\{nC_{0}+n_{1}C_{1}+r_{1}C_{2}+r_{2}C_{3}\}\\ & =n\{C_{0}+E(\frac{n_{1}}{n})C_{1}+E(\frac{r_{1}}{n})C_{2}+E(\frac{r_{2}}{n})C_{3}\}\\ & =\frac{n}{N}\{NC_{0}+N_{1}C_{1}+\frac{1}{k}(R_{1}C_{2}+R_{2}C_{3})\}\\ & =n\{C_{0}+W_{1}C_{1}+\frac{1}{Nk}C_{\left(2\right)}\} \end{array}$$ (34)

So, we have the following Lemma, as:

**Lemma 4.1**. The optimum values of *k* and *n* for the minimum expected cost are, respectively, given by

$$\begin{array}{cc} k_{ov} & =\sqrt{\frac{C_{\left(2\right)}({\bar{Y}}^{2}C_{u_{}}^{2}+\frac{1}{Nr_{1}}\sum_{i=1}^{N_{2}}\phi_{i}-W_{2}{\bar{Y}}_{2}^{2}C_{g_{\left(2\right)}}^{2*})}{N(C_{0}+W_{1}C_{1})W_{2}{\bar{Y}}_{2}^{2}C_{g_{\left(2\right)}}^{2*}}} \end{array}$$ (35)

and

$$\begin{array}{c} n_{ov}=\frac{{\bar{Y}}^{2}C_{u_{}}^{2}+\frac{1}{Nr_{1}}\sum_{i=1}^{N_{2}}\phi_{i}+W_{2}\left(k-1\right){\bar{Y}}_{2}^{2}C_{g_{\left(2\right)}}^{2*}}{V_{0}+\frac{{\bar{Y}}^{2}C_{y_{}}^{2}}{N}}, \end{array}$$ (36)

where *g* = *c*, *d*, and *p*.

**Proof**. Let the variance $V({\hat{\bar{u}}}_{\left(g\right)})$ be a fixed *V*_0_, i.e $V({\hat{\bar{u}}}_{\left(g\right)})=V_{0}$, then the Lagrange function, is given by

$$\begin{array}{cc} L & =n\{C_{0}+W_{1}C_{1}+\frac{1}{Nk}C_{\left(2\right)}\}+\xi \{V{\left({\hat{\bar{u}}}_{\left(g\right)}\right)}_{\text{min.}}-V_{0}\}, \end{array}$$ (37)

where *ξ* is a Lagrange multiplier. Differentiating (37) with respect to *n*, equating to zero i.e $\left(\frac{\partial L}{\partial n}=0\right)$ and ignoring *δ*_*j*_, *j* = (1or2). We have

$$\begin{array}{c} \frac{\partial L}{\partial n}=C_{0}+W_{1}C_{1}+\frac{1}{Nk}C_{\left(2\right)}+\frac{\xi }{n^{2}}\{-{\bar{Y}}^{2}C_{u_{}}^{2}-\frac{1}{Nr_{1}}\sum_{i=1}^{N_{2}}\phi_{i}-W_{2}(k-1){\bar{Y}}_{2}^{2}C_{g_{\left(2\right)}}^{2*}\}=0 \end{array}$$

which implies

$$\begin{array}{c} n=\frac{\sqrt{\xi }\sqrt{N\{{\bar{Y}}^{2}C_{u}^{2}+\frac{1}{Nr_{1}}\sum_{i=1}^{N_{2}}\phi_{i}+W_{2}(k-1){\bar{Y}}_{2}^{2}C_{g_{\left(2\right)}}^{2*}\}}}{\sqrt{N(C_{0}+W_{1}C_{1}+\frac{1}{Nk}C_{\left(2\right)})}} \end{array}$$ (38)

Note that

$$\begin{array}{cc} V{\left({\hat{\bar{u}}}_{\left(g\right)}^{*}\right)}_{\text{min.}}≅ & \frac{1}{Nnr_{1}}\sum_{i=1}^{N_{2}}\phi_{i}+\frac{1}{n}W_{2}\left(k-1\right){\bar{Y}}_{2}^{2}C_{g_{\left(2\right)}}^{2*}+(\frac{1}{n}-\frac{1}{N}){\bar{Y}}^{2}C_{u_{}}^{2} \end{array}$$ (39)

Substituting (38) in (39), we have

$$\begin{array}{c} \sqrt{\xi }=\frac{\sqrt{N(C_{0}+W_{1}C_{1}+\frac{1}{Nk}C_{\left(2\right)})}\sqrt{{\bar{Y}}^{2}{C_{u_{}}}^{2}+\frac{1}{Nr_{1}}\sum_{i=1}^{N_{2}}\phi_{i}+W_{2}(k-1){\bar{Y}}_{2}^{2}C_{g_{\left(2\right)}}^{2*}}}{\sqrt{N}(V_{0}+\frac{1}{N}{\bar{Y}}^{2}C_{u}^{2})} \end{array}$$ (40)

Substituting (40) in (38), we have

$$\begin{array}{c} n_{ov}=\frac{{\bar{Y}}^{2}C_{y_{}}^{2}+\frac{1}{Nr_{1}}\sum_{i=1}^{N_{2}}\phi_{i}+W_{2}(k-1){\bar{Y}}_{2}^{2}C_{g_{\left(2\right)}}^{2*}}{V_{0}+\frac{{\bar{Y}}^{2}C_{u}^{2}}{N}} \end{array}$$ (41)

which is the required optimum sample size (*n*_*ov*_). Now, we differentiate (37) with respect to *k* and equate to zero i.e $\left(\frac{\partial L}{\partial k}=0\right)$. Then, we have

$$\begin{array}{c} \frac{\partial L}{\partial k}=\xi \frac{1}{n}W_{2}{\bar{Y}}_{2}^{2}C_{g_{\left(2\right)}}^{2*}-\frac{1}{Nk^{2}}C_{\left(2\right)}=0 \end{array}$$

which implies

$$\begin{array}{c} k^{2}=\frac{nC_{\left(2\right)}}{N\xi \;W_{2}{\bar{Y}}_{2}^{2}C_{g_{\left(2\right)}}^{2}} \end{array}$$ (42)

Using (38) in (42), we have

$$\begin{array}{cc} k_{ov} & =\sqrt{\frac{C_{\left(2\right)}({\bar{Y}}^{2}C_{u_{}}^{2}+\frac{1}{Nr_{1}}\sum_{i=1}^{N_{2}}\phi_{i}-W_{2}{\bar{Y}}_{2}^{2}C_{g_{\left(2\right)}}^{2*})}{N(C_{0}+W_{1}C_{1})W_{2}{\bar{Y}}_{2}^{2}C_{g_{\left(2\right)}}^{2*}}} \end{array}$$ (43)

which is the required optimum value of *k*.

**Corollary 4.1.1**. The optimum values of *n* and *k* are proportional to the expected cost (C*). To get optimum values of *k* and *n*, that, $V{\left({\hat{\bar{u}}}_{\left(g\right)}^{*}\right)}_{\text{min.}}$, we simply substitute $C_{y_{}}^{2}$ and $C_{g_{\left(2\right)}}^{2}$ in (35) and (36).

## 5 Empirical comparison

On the lines of [25], the relative comparison of ${\hat{\bar{u}}}_{\left(q\right)}^{*}$ with respect to ${\hat{\bar{u}}}_{}^{*}$ is considered by generating a hypothetical population under following key steps, as:

1. Let two independent populations say {Ω_(*x*)_ and Ω_(*z*)_} of size 1000 are obtained from gamma distribution, using following parametric values, as:
  γx=(1.55.5)andγz=(1.54.5) (44)
  The study variable is generated by
  $$\begin{array}{cc} y_{i} & =2.8+\sqrt{\left(1-0.2\right)}z_{i}+x_{i},\;\rho_{uv}=0.85 \end{array}$$ (45)
2. Splitting the populations into two strata having *N*_1_ = 690 and *N*_2_ = 310 units.
3. Assume that, out of *N*_2_ units, *R*_1_ units provides the response by using (5) and remaining *R*_2_ = (*N*_2_ − *R*_1_) are those who refuse to give their true or scrambled responses.
4. Imputing the missing *R*_2_ values by using $\bar{X}$ and *S*_*uv*_.
5. Repeat the process H=(Nnov) times. The variance of the given estimator is obtained by using following expression, as:
  $$\begin{array}{c} V({\hat{\bar{u}}}_{\left(g\right)}^{*})=\frac{1}{H}\sum_{l=1}^{H}{\left\{{\hat{\bar{u}}}_{\left(v\right)}^{*}\left(l\right)-\bar{Y}\right\}}^{2} \end{array}$$ (46)
  and the relative efficiency (R.E) of ${\hat{\bar{u}}}_{\left(g\right)}^{*}$ is obtained by using the following expression
  $$\begin{array}{c} R.E\left(j\right)=\frac{V({\hat{\bar{u}}}_{}^{*})}{V({\hat{\bar{u}}}_{\left(g\right)}^{*})}\;\text{for}\;j=1,2,\;\text{and}\;3. \end{array}$$ (47)

For the numerical comparison, we consider the following values of un-known constants, as:

$$\begin{array}{c} C^{*}=200,\;C_{o}=20,\;C_{1}=50,\;C_{\theta_{1}}=C_{\theta_{2}}=10,\;{\left(\frac{\theta_{2}}{\theta_{1}}\right)}^{2}=0.1,\;\text{and}\;r=0.4\;n_{og}, \end{array}$$

where *r* is assumed response rate, which is 40% of *n*_*og*_. The optimum values of relative efficiencies (R.Es) of ${\hat{\bar{u}}}_{\left(g\right)}^{*}$ are given in Table 1.

**Table 1:** Optimum values of *k*, *n*, and R.E(*j*) w.r.t. ${\hat{\bar{u}}}_{}^{*}$.

*C*_2_	*C*_3_	*r*_1_	*V*_*o*_	*k*_*op*_	*n*_*op*_	Relative Efficiency		
						R.E(1)	R.E(2)	R.E(3)
1	2.0	10	4	2.30	191	1.48	2.08	3.83
1	2.0	20	8	1.63	58	1.75	2.72	3.88
1	2.0	30	12	1.34	28	1.91	3.41	3.96
1	1.0	10	4	1.93	191	1.34	2.08	3.37
1	1.0	20	8	1.37	58	1.93	3.01	3.64
1	1.0	30	12	1.12	28	1.64	2.42	3.91
1	0.5	10	4	1.71	191	1.54	1.67	3.68
1	0.5	20	8	1.21	58	1.56	2.88	4.01
1	0.5	30	12	1.09	28	1.22	1.95	4.52
2	2.0	10	4	2.68	191	1.67	2.40	3.89
2	2.0	20	8	1.90	58	1.88	3.39	4.45
2	2.0	30	12	1.56	28	2.49	3.57	4.89
2	1.0	10	4	2.38	191	1.57	1.85	3.29
2	1.0	20	8	1.69	58	1.26	2.91	3.90
2	1.0	30	12	1.38	28	1.57	2.85	4.04
2	0.5	10	4	2.29	191	1.59	2.23	3.06
2	0.5	20	8	1.57	58	1.47	2.74	3.19
2	0.5	30	12	1.29	28	1.33	2.65	3.45
3	2.0	10	4	2.99	191	1.36	1.86	2.44
3	2.0	20	8	2.12	58	1.59	2.28	3.22
3	2.0	30	12	1.74	28	1.85	2.61	4.02
3	1.0	10	4	2.74	191	1.48	2.59	2.74
3	1.0	20	8	1.94	58	1.66	2.97	3.07
3	1.0	30	12	1.59	28	1.84	3.72	4.36
3	0.5	10	4	2.61	191	1.61	2.49	3.36
3	0.5	20	8	1.85	58	1.78	3.16	3.54
3	0.5	30	12	1.51	28	2.33	4.11	4.31

Table 1 shows the optimum values of *k*, *n*, and R.E(j) of estimators i.e modified ratio and difference estimators. Under this hypothetical population, the modified estimators i.e ${\hat{\bar{u}}}_{\left(g\right)}^{*}$ perform better as compared to traditional Hansen-Hurwitz estimator $\left({\hat{\bar{u}}}^{*}\right)$. We also observe that the optimum value of *n*_*og*_ is approximately similar for all ${\hat{\bar{u}}}_{\left(p\right)}^{*}$, so optimum sample of size *n*_*op*_ is used for the relative comparison between existing and proposed imputation estimators.

From Table 1, we observed following proportionality relationships between *C*_2_, *C*_3_, *r*_1_, *V*_*o*_, *k*_*op*_, *n*_*op*_, and R.E(*j*).

1. The values of *V*_*o*_ and *n*_*op*_ have inverse relationship with *C*_2_ and *C*_3_.
2. *C*_2_ and *C*_3_ have the positive relationship with RE(*j*). As the costs of scrambling response and imputation increase, the relative efficiencies of ${\hat{\bar{u}}}_{\left(g\right)}^{*}$ have been improved significantly.
3. *r*_1_ has the negative association with *k*_*op*_ and *n*_*op*_. The values of *k*_*op*_ and *n*_*op*_ decrease as *r*_1_ increasing.
4. *V*_*o*_ also has the inverse relationship with *k*_*op*_ and *n*_*op*_. As the value of *V*_*o*_ increases, the values of *k*_*op*_ and *n*_*op*_ decrease.
5. The relative efficiencies of ${\hat{\bar{u}}}_{\left(g\right)}^{*}$ are also inversely correlated with *r*_1_ and *V*_*o*_. The values of R.E(*j*) decrease as *V*_*o*_ and *n*_*op*_ increase.

From the numerical finding, we can conclude that the proposed imputation procedure at second call should be performs better as compared to existing and tradition Hansen-Hurwitz estimators at various values of *C*_2_, *C*_3_, *r*_1_ and *V*_*o*_.

## 6 Conclusion

The problem of non-response bias in the sensitive quantitative study variable has been diminished by sub-sampling the non-respondent, viz. Hansen and Hurwitz (1946) procedure. A new imputation mechanism has been defined by using the known co-variance between the study variable and the auxiliary variable. Optimum value for sample size is also derived for a given set of unit cost (*C*_*q*_, *q* = 0, 1, 2, 3), *r*_1_, and *V*_*o*_. From the Table 1, we can easily say that the proposed imputation method can outperforms as compared to ratio, difference, and Hansen-Hurwitz estimators.

When the processing, editing, or imputing cost per unit is high, the proposed imputation strategy can performs better as compared to their counterpart. Our proposed imputation procedure is also useful when there are serious concerns about the non-response bias or refusals due to the sensitive nature of the study variable that is difficult to ignore it.

## Supporting information

S1 Code In this research a hypothetical data set is used which can be easily regenerated at the given value of parameters with the help of available statistical software. (R)
